# Comparison of subclavian vein to inferior vena cava collapsibility by ultrasound in acute heart failure: A pilot study

**DOI:** 10.1002/clc.23758

**Published:** 2021-12-21

**Authors:** Yvonne E. Kaptein, Elaine M. Kaptein

**Affiliations:** ^1^ Aurora Cardiovascular and Thoracic Services Aurora Sinai/Aurora St. Luke's Medical Centers, Advocate Aurora Health Milwaukee Wisconsin USA; ^2^ Department of Medicine, Division of Nephrology University of Southern California Los Angeles California USA

**Keywords:** acute decompensated heart failure, inferior vena cava ultrasound, subclavian/proximal axillary vein ultrasound, tricuspid regurgitation

## Abstract

**Background:**

Management of acute decompensated heart failure (ADHF) requires accurate assessment of relative intravascular volume, which may be technically challenging. Inferior vena cava (IVC) collapsibility with respiration reflects intravascular volume and right atrial pressure (RAP). Subclavian vein (SCV) collapsibility may provide an alternative.

**Hypothesis:**

The purpose of this study was to examine the relationship between SCV collapsibility index (CI) and IVC CI in ADHF.

**Methods:**

This was a prospective study of non‐ventilated patients with ADHF who had paired IVC and SCV ultrasound assessments. As SCV CI is highly position‐dependent, measurements were performed supine at 30–45°.

**Results:**

Thirty‐three patients were included with 36 encounters. The sample size was adequately powered for receiver‐operator characteristic (ROC) analysis. SCV CI correlated with IVC CI during relaxed breathing (*R* = .65, *n* = 36, *p* < .001) and forced inhalation (*R* = .47, *n* = 36, *p* = .0036). SCV CI < 22% and >33% corresponded to IVC CI < 20% and >50% suggesting hypervolemia (sensitivity/specificity: 72%) and hypovolemia (sensitivity/specificity: 78%), respectively. Moderate to severe tricuspid regurgitation (TR) compared to less than moderate TR was associated with lower SCV CI (medians: 12.4% vs. 25.3%, *p* = .022) and IVC CI (medians: 9.6% vs. 35.6%, *p* = .0012). SCV CI and IVC CI were not significantly different among chronic kidney disease stages.

**Conclusion:**

In non‐ventilated ADHF, SCV CI at 30–45° correlates with paired IVC CI, and may provide an alternative to IVC CI for assessment of relative intravascular volume, which may facilitate clinical management. Moderate to severe TR decreases SCV CI and IVC CI and may result in overestimation of relative intravascular volume.

**Abbreviations**
ADHF: acute decompensated heart failure
CI: collapsibility index
IQR: interquartile range
IVC: inferior vena cava
JVP: jugular venous pressure
NT‐proBNP: N‐terminal prohormone of brain natriuretic peptide
RAP: right atrial pressure
SCV: subclavian vein
TR: tricuspid regurgitation

## 1 INTRODUCTION

Early, accurate, and ongoing intravascular volume assessment in patients admitted with heart failure is required to provide effective goal‐directed therapy, minimize adverse outcomes of inappropriate, ineffective, or excessive diuresis, and improve survival. Although 65% of nonedematous patients with chronic heart failure are intravascularly hypervolemic, 30% are euvolemic, and 5% are hypovolemic.^1^ Assessment of relative intravascular volume by physical examination may be inaccurate,^1^, ^2^ and N‐terminal prohormone of brain natriuretic peptide (NT‐proBNP) is nonspecific for volume overload.^1^

The American Society of Echocardiography supports the use of ultrasound assessment of IVC maximum diameter (IVC_max_) and IVC collapsibility index (CI) with respiration to estimate right atrial pressure (RAP),^3^, ^4^ with recent validation.^5^ IVC CI measurements have reasonable sensitivity (84%–96%) and specificity (37%–93%) for diagnosis of congestive heart failure,^6^ and may help guide diuresis and heart failure therapy to improve patient outcomes.^7^

The goal of volume management is to improve cardiac output by optimizing relative intravascular volume. The gold standard for assessing volume responsiveness has been defined as an increase in cardiac output of ≥10% in response to volume administration.^2^, ^8^, ^9^ Dynamic parameters that take into account respiratory variation, such as IVC CI^10^, ^11^ and subclavian vein (SCV) CI,^12^ have the best sensitivity and specificity for estimating relative intravascular volume and predicting response to volume administration. These dynamic parameters are objective measures of the jugular venous waveform, with increased respiratory variation suggesting intravascular hypovolemia or volume responsiveness.

Ultrasound measurements of IVC CI have been used to assess relative intravascular volume overload in patients with congestive heart failure^6^, ^7^, ^13^, ^14^, ^15^ and/or renal failure^16^, ^17^ and to predict which patients may benefit from volume removal. An IVC CI of <20% was related to the ability to remove volume by ultrafiltration^16^ and/or increase cardiac output after volume removal^17^ in renal failure. In patients with shortness of breath due to various etiologies, IVC CI was greater in nonheart failure (46%) than in heart failure (9.6%) patients (*p* < .0001).^13^ At an IVC CI cutoff of 15%, sensitivity and specificity for the diagnosis of congestive heart failure were 92% and 84%, respectively.^13^ IVC diameter and variation with respiration may be altered by changes in relative intravascular volume as well as by moderate to severe tricuspid regurgitation (TR).^15^, ^18^, ^19^, ^20^

SCV CI has been studied as an alternative assessment of relative intravascular volume, as IVC imaging can be technically challenging.^21^ SCV CI measured with the patient reclining at a 30° angle predicted volume responsiveness in mechanically ventilated critically ill patients.^12^ SCV CI measured in patients reclining at 30–45° correlated well with subcostal IVC CI in patients undergoing echocardiography,^22^ in patients treated in the surgical intensive care unit ^21^ and in those hospitalized with acute and/or chronic renal failure.^23^ SCV CI has not previously been compared to IVC CI in patients hospitalized with acute congestive heart failure.

The purpose of this study is to compare point‐of‐care ultrasound of SCV to IVC as an assessment of relative intravascular volume in patients hospitalized with acute exacerbation of heart failure.

## 2 METHODS

This prospective study received institutional review board approval at Aurora St. Luke's Medical Center in Milwaukee, Wisconsin (IRB 19‐135ET). Adult patients hospitalized with heart failure exacerbation requiring intravenous diuretics were recruited from January 13, 2020, to March 6, 2020. All patients who met inclusion criteria without meeting exclusion criteria, and who signed a written consent were evaluated. All had NT‐proBNP measured on admission. Patients were excluded if they were mechanically ventilated or on noninvasive positive pressure ventilation; unable to give consent; non‐English speaking; postheart transplantation; currently had a left ventricular assist device, Impella, or intra‐aortic balloon pump; or declined participation. Patients with mechanical circulatory support were excluded due to concerns about effects on hemodynamics. Patients with right‐sided devices and/or with TR were included to assess how TR affects IVC CI and SCV CI. TR was quantified based on the formal echocardiogram closest to the time of the recruitment.

Patient assessment was conducted within 24 h of routine admission NT‐proBNP. Jugular venous pressure (JVP) was measured supine at an incline of at least 30°. The severity (0–4+) of lower extremity pitting edema was assessed.

**Figure 1:**
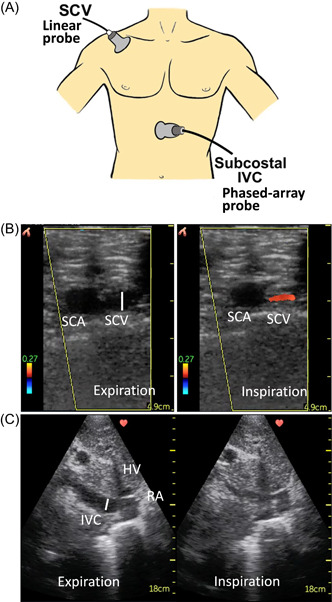
Ultrasound probe positions and ultrasound images of the subclavian vein (SCV) and inferior vena cava (IVC) with respiration. (A) Ultrasound probe positions: distal SCV images were obtained with patients lying supine at an incline of 30–45°. A linear array transducer was placed inferior to the lateral border of the right clavicle, aligned in the deltopectoral groove along the axis of the right arm to obtain a transverse view of the SCV at the junction with the proximal axillary vein. The IVC was imaged via the subcostal window in a longitudinal plane using the phased array transducer. (B) Ultrasound images of the subclavian artery (SCA) and SCV using the linear array transducer with color‐flow Doppler to ensure imaging of the appropriate vessel and to clarify SCV borders. (C) Ultrasound images of the IVC using the phased array transducer. Images are shown during expiration and inspiration. Maximum and minimum SCV and IVC diameters were measured perpendicular to the inner edge of the vessel walls as illustrated by the white lines. IVC diameters were measured 2 cm from the right atrium (RA) or distal to the hepatic vein (HV)

Ultrasound images and measurements were obtained using a handheld portable VScan with Dual Probe (GE Healthcare), with patients supine at an incline of 30–45°. The distal subclavian/proximal axillary vein^24^ was imaged using the linear array transducer with color‐flow Doppler to ensure imaging of the appropriate vessel and to clarify vessel borders (Figure 1). The probe was placed inferior to the lateral border of the right clavicle, aligned in the deltopectoral groove along the axis of the right arm to obtain a transverse view of the SCV at the junction of the proximal axillary vein (Figure 1A).^21^, ^23^, ^24^ The IVC was imaged via the subcostal window in a longitudinal plane using the phased array transducer (Figure 1A). For both IVC and SCV, maximum (*D*_max_) and minimum (*D*_min_) diameters were measured with both relaxed breathing and forced inspiration or “sniff.” A frame‐by‐frame analysis of greyscale (B‐Mode) images was performed to identify *D*_max_ and *D*_min_, which were then measured using the digital calipers (GE Healthcare) (Figures 1B,C).^25^, ^26^

$$\text{Collapsibility}\;\text{index}\;\text{was}\;\text{calculated}\;\text{as}\;\text{CI}=\left[\left(D_{\max}–D_{\min}\right)/D_{\max}\right]\times 100\%.$$

For three patients, physical examination and bedside ultrasound measurements were repeated on subsequent days, within 24 h of repeat NT‐proBNP.

Physical examination and bedside ultrasound were conducted by the first author. Ultrasound studies of five patients were independently performed by a critical care attending, and measurements were shown to agree with those of the first author. The majority of stored deidentified ultrasound images were independently reviewed for measurement accuracy by the senior author who has extensive ultrasound experience. Encounters were excluded if either the SCV or IVC were not adequately visualized.

After the initial assessment, each patient's chart was reviewed by the first author for further data collection (Table 1). Data were presented as median ± interquartile ranges, as data were not normally distributed. Pearson's correlation coefficient was calculated to assess the relationship between SCV CI and IVC CI, with both relaxed breathing and forced inhalation. Bland–Altman analysis was used to examine whether there was systematic bias between SCV CI and IVC CI values. Mann–Whitney rank‐sum test was used to determine whether two groups have the same numerical values for a given variable. Kruskal–Wallace rank‐sum test examines whether two or more groups have the same numerical values, and if significant, Duncan's multiple comparisons test was performed.

**Table 1:** Patient characteristics

Characteristic	*n* = 33 (unique patients)		
Age, years	66 (60–76)		
Female	16 (48%)		
Male	17 (52%)		
Race			
African American	10 (30%)		
White	21 (64%)		
Other	2 (6%)		
BMI, kg/m^2^ (at time of dry weight)	28.1 (24.1–36.5)		
History of HTN	30 (91%)		
History of CAD	18 (55%)		
Prior stents	7		
Prior CABG	8		
History of PAD/PVD	5 (15%)		
History of CKD	21 (64%)		
CKD Stage 2	1		
CKD Stage 3	14		
CKD Stage 4	5		
CKD Stage 5/ESRD	1		
History of DM	12 (36%)		
History of AFib/AFL^a^	18 (55%)		
History of OSA	9 (27%)		
History of valvular disease^b^	19 (58%)		
Historical heart failure diagnosis	30 (91%)	Lowest lifetime EF before admission	Most recent EF before admission
HFrEF (LVEF < 40%)	20 (69%)	21.9% ± 8.0%	23.4% ± 7.6%
HFmrEF (LVEF 40%–49%)	4 (14%)	43.5% ± 2.1%	45.0% ± 2.5%
HFpEF (LVEF ≥ 50%)	6 (18%)	56.5% ± 2.8%	58.9% ± 5.4%
LVEF (most recent before admission)	38.0% (23.0%–51.0%)		
Type of cardiomyopathy (for HFrEF/HFmrEF)			
Ischemic	11 (46%)		
Nonischemic	11 (46%)		
Mixed	2 (8%)		
Devices (PPM and/or ICD)	11 (33%)		
NYHA functional class (before admission)			
I	2		
II	7		
III	12		
IV	7		
Not reported	5		
NT‐proBNP on admission (pg/ml)	5731 (2968–13277)		
Troponin on admission (ng/ml)	<0.10 (<0.05–0.13)		
Loop diuretic infusion received	18 (55%)		
Inotrope or pressor received	9 (27%)		
Differences between admission and discharge values			
Weight, kg	−6.9 (−11.3 to −2.2)		
Serum creatinine, mg/dl (*n* = 33)	0.0 (−0.11 to +0.30)		
NT‐proBNP, pg/ml (*n* = 23)	−216 (−5303 to +680)		
NT‐proBNP, percent change	−10.1% (−59.8% to +32.7%)		
Net intake minus output, ml	−6485 (−12256 to −3140)		
Hospital length of stay (days)	8 (5–12)		
All‐cause 30‐day readmission or ED visit (unplanned)	10 (31%)		
Mortality after 12 months^c^	10 (30%)		

*Note*: Data presented as *n* (%), median (interquartile range), or mean ± standard deviation.

Abbreviations: AFib, atrial fibrillation; AFL,  atrial flutter; BMI,  body mass index; CABG,  coronary artery bypass graft; CAD,  coronary artery disease; CKD,  chronic kidney disease; DM,  diabetes mellitus; ED,  emergency department; HFmrEF, heart failure with mid‐range ejection fraction; HFpEF, heart failure with preserved ejection fraction; HFrEF, heart failure with reduced ejection fraction; HTN,  hypertension; ICD,  implantable cardioverter‐defibrillator; LVEF,  left ventricular ejection fraction; NT‐proBNP, N‐terminal prohormone of brain natriuretic peptide; NYHA,  New York Heart Association; OSA,  obstructive sleep apnea; PAD,  peripheral arterial disease; PPM,  permanent pacemaker; PVD,  peripheral vascular disease.

^a^ All patients with atrial fibrillation or atrial flutter were rate controlled. A recent article by Berthelot et al.^27^ reported that IVC_max_ and IVC CI were valuable to identify patients with HFpEF with high left ventricular filling pressures even in patients with sinus rhythm or atrial fibrillation.

^b^ Valvular heart disease is defined as at least moderate level regurgitation, at least mild level stenosis, or history of valve repair or replacement.

^c^ 12 months from the date of patient enrollment.

Sensitivity and specificity curves were generated using SigmaPlot version 13 (Systat Software Inc.) to assess SCV CI cutoff values which predict IVC CI < 20% suggesting hypervolemia (RAP ≥ 20 mmHg), or IVC CI > 50% suggesting hypovolemia (RAP < 5 mmHg) as the reference standards. We calculated receiver operator characteristic curves and areas under the curve from observed sensitivity and specificity data.

The IVC CI cutoff of <20% likely excludes hypovolemia and thus may indicate hypervolemia. This cutoff was derived from the ability to remove various volumes of ultrafiltrate during hemodialysis,^16^ and is further endorsed by the 2010 American Society of Echocardiography guidelines, which state that for those patients unable to adequately perform a sniff, an IVC CI < 20% with quiet inspiration suggests elevated RAP, and hence hypervolemia.^3^ The IVC CI cutoff of >50% likely excludes hypervolemia and thus indicates possible hypovolemia. This was derived from prior publications comparing paired IVC CI to RAP by right heart catheterization.^14^, ^16^, ^28^, ^29^, ^30^ In these publications, an RAP of ≥20 mmHg was associated with IVC CI < 20% in 88% of encounters, and RAP of <5 mmHg was associated with IVC CI > 50% in 80% of encounters.^16^

The sample size was based on the time available to recruit patients for this project, which is a pilot study. Subsequently, power analysis for the data obtained was used to determine whether the study was sufficiently powered.^31^, ^32^

## 3 RESULTS

**Figure 2:**
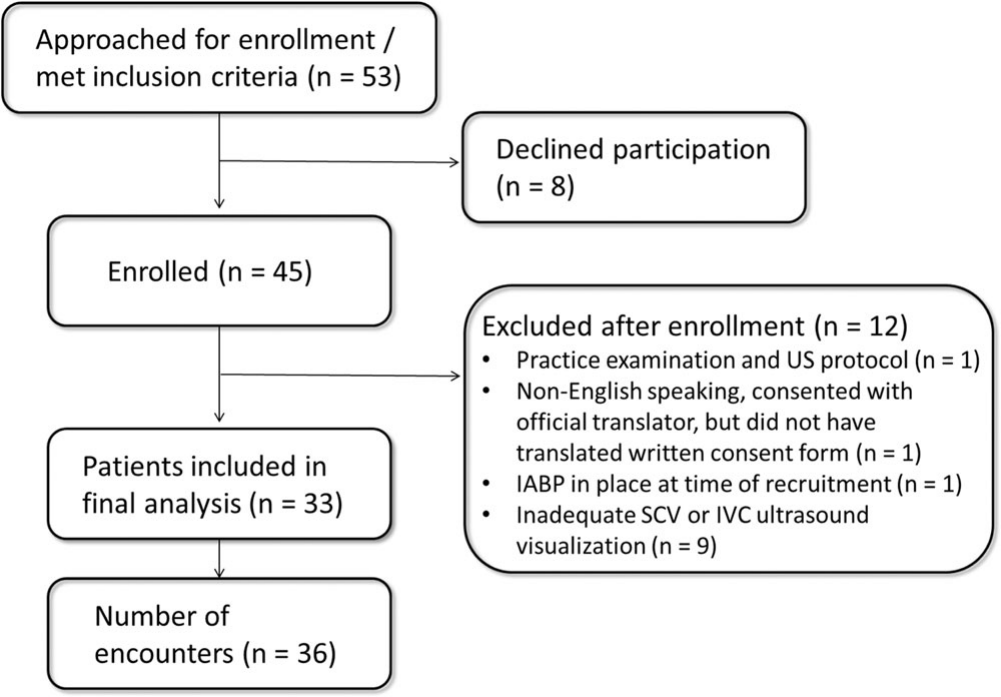
Patient recruitment. IABP, intra‐aortic balloon pump; IVC, inferior vena cava; SCV, subclavian vein; US, ultrasound

As shown in Figure 2, 33 patients were included, with 36 unique patient encounters. Three patients were examined and scanned twice during the same admission.

Patient characteristics are shown in Table 1.

**Figure 3:**
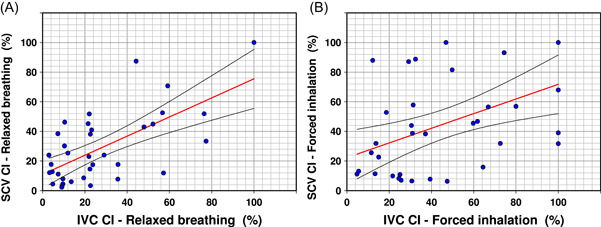
Correlations of SCV CI to IVC CI with both relaxed breathing and forced inhalation. Black curved lines represent 95% confidence interval. (A) Correlation of SCV CI to IVC CI with relaxed breathing. *R* = .65, *n* = 36, *p* < .001. (B) Correlation of SCV CI to IVC CI with forced inhalation. *R* = .47, *n* = 36, *p* = .0036. CI, collapsibility index; IVC, inferior vena cava; SCV, subclavian vein

The paired SCV and IVC collapsibility indices were significantly correlated during relaxed breathing (*R* = .65, *n* = 36, *p* < .001, Figure 3A) and during forced inhalation (*R* = .47, *n* = 36, *p* = .0036, Figure 3B). Bland–Altman analysis indicated that there was no systematic bias between SCV and IVC collapsibility. Due to the inferior correlation with forced inhalation, only relaxed breathing was considered for subsequent analyses.

**Figure 4:**
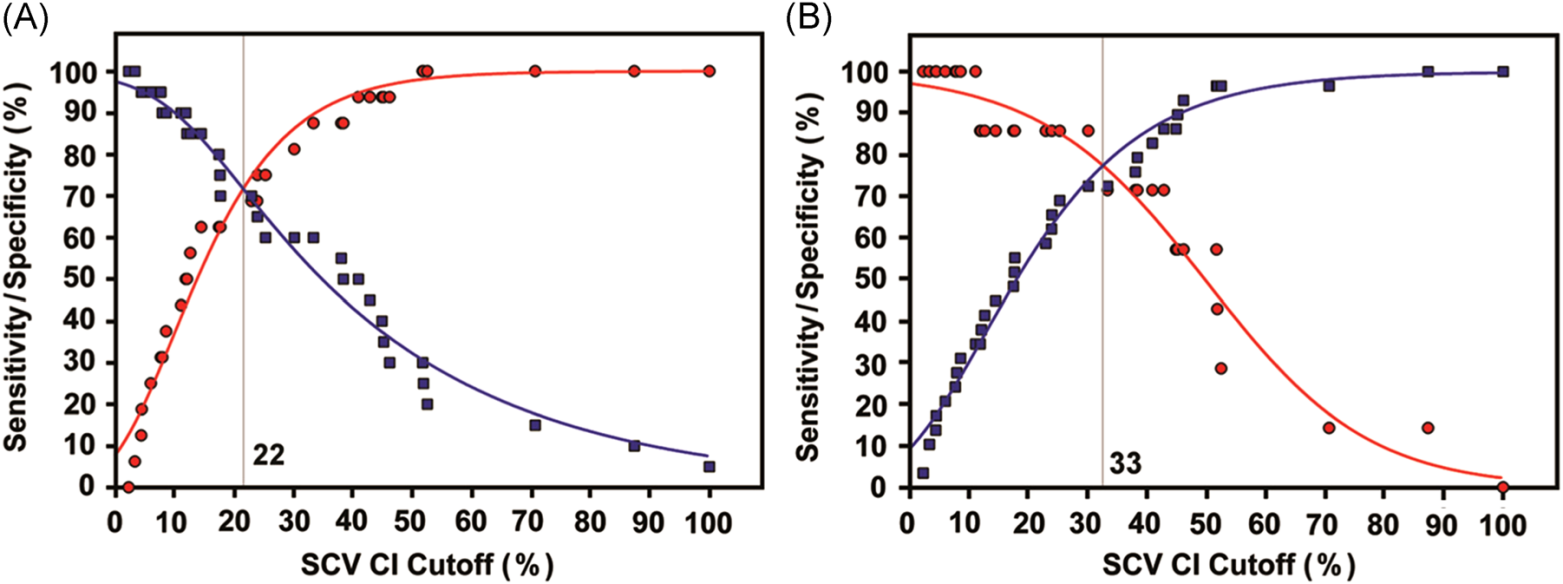
Sensitivity and specificity for SCV CI cutoffs, as predictors for IVC CI < 20% or >50%, with relaxed breathing. Red circles represent sensitivity and blue squares specificity. Solid curves are sigmoidal fit to data, with values for sensitivity and specificity maxima and minima constrained to 100% and 0%, respectively. SCV CI cutoffs at which sensitivity and specificity are equal and maximal are indicated by the vertical line. (A) Sensitivity and specificity for SCV CI cutoffs, as predictors for whether IVC CI is <20%, suggesting hypervolemia. The SCV CI cutoff of <22% corresponded to equivalent sensitivity/specificity of 72% (AUC of ROC plot = 0.786 ± 0.076 [SE], *n* = 36, *p* = .000085). (B) Sensitivity and specificity for SCV CI cutoffs, as predictors for whether IVC CI is >50%, suggesting hypovolemia. SCV CI cutoff of >33% corresponded to equivalent sensitivity/specificity of 78% (AUC of ROC plot = 0.833 ± 0.091 [SE], *n* = 36, *p* = .000127). AUC, area under the curve; CI, collapsibility index; IVC, inferior vena cava; ROC, receiver‐operator characteristic; SE, standard error; SCV, subclavian vein

Based on prior studies, IVC CI > 50% and <20% have been shown to correspond to RAP by right heart catheterization of <5 mmHg and >20 mmHg, respectively.^16^, ^23^ Sensitivity and specificity of SCV CI cutoffs predicting these specific IVC CI cutoffs are shown in Figure 4. At all possible discrimination thresholds for SCV CI, sensitivities and specificities for whether SCV CI below the cutoff predicts IVC CI < 20% are shown in Figure 4A, AND whether SCV CI greater than the cutoff predicts IVC CI > 50% are shown in Figure 4B. SCV CI of <22% best predicted IVC CI < 20% (unlikely hypovolemia), with sensitivity/specificity of 72%. SCV CI > 33% best predicted IVC CI > 50% (unlikely hypervolemia), with sensitivity/specificity of 78%.

For the determination of the SCV CI cutoff which predicts IVC CI of <20%, there were 16 encounters with IVC CI < 20% and 20 encounters with IVC CI > 20%. This number is sufficient at *α* = .05 and *β* = .2 for receiver‐operator characteristic (ROC) analysis provided area under the curve (AUC) is 0.73 or greater. For our study, AUC was 0.786; thus the study was sufficiently powered to conclude that an SCV CI cutoff of <22% correctly predicts the IVC CI cutoff of <20%.

For the determination of the SCV CI cutoff which predicts IVC CI of >50%, there were 7 encounters with IVC CI > 50% and 29 encounters with IVC CI < 50%. This number is sufficient at *α* = .05 and *β* = .2 for ROC analysis provided AUC is 0.78 or greater. For our study, AUC was 0.833; thus the study was sufficiently powered to conclude that an SCV CI cutoff of >33% correctly predicts the IVC CI cutoff of >50%.

Eighteen patients had at least moderate severity TR, 11 had right‐sided intracardiac devices, and, of these, seven had both. We examined the possible effect of at least moderate severity TR and of right‐sided intracardiac devices (i.e., permanent pacemaker or implantable cardioverter‐defibrillator wires) on SCV diameters.

**Figure 5:**
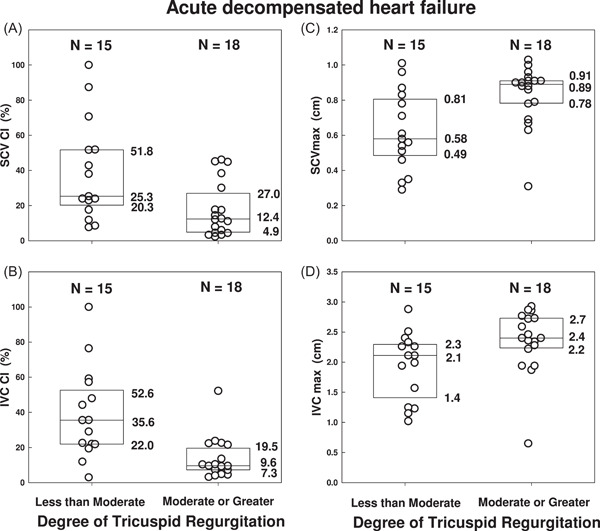
Relationship of first encounter SCV and IVC collapsibility indices and maximum diameters to the severity of tricuspid regurgitation in acute decompensated heart failure. Panels A and B show the relationship of SCV CI and IVC CI to TR in the current study population with acute decompensated heart failure. Higher SCV CI (*p* = .022 Mann–Whitney) and IVC CI (*p* = .0012 Mann–Whitney) were seen with less than moderate TR, compared to moderate or greater TR. Panels C and D show the effect of TR on SCV_max_ and IVC_max_. Lower SCV_max_ (*p* = .00097 Mann–Whitney) and IVC_max_ (*p* = .026 Mann–Whitney) were seen with less than moderate TR, compared to moderate or greater TR in these patients with acute decompensated heart failure. Boxes represent medians and interquartile ranges. CI, collapsibility index; IVC, inferior vena cava; SCV, subclavian vein; TR, tricuspid regurgitation

As shown in Figure 5A, for the first encounter used for each patient, moderate to severe TR was associated with significantly lower median SCV CI (12.4%) compared to mild or no TR (25.3%, *p* = .022). Likewise, median IVC CI (Figure 5B) was significantly lower with moderate to severe TR (9.6%) compared to mild or no TR (35.6%, *p* = .0012).

As shown in Figure 5C, median SCV_max_ was significantly larger with moderate to severe TR (0.89 cm) compared to less than moderate TR (0.58 cm, *p* = .00097). Median IVC_max_ with moderate to severe TR was also significantly larger (2.4 cm) than with less than moderate TR (2.1 cm, *p* = .026) (Figure 5D).

The presence or absence of right‐sided intracardiac devices was not associated with differences in SCV CI (*p* = .67 Mann‐Whitney) or IVC CI (*p* = .54 Mann–Whitney).

Although 21 patients had chronic kidney disease (CKD), admission NT‐proBNP levels were not significantly different among those without CKD, with CKD Stages 2–3, or with CKD Stages 4–5 (*p* = .368 by Kruskal–Wallis). Nor were SVC CI (*p* = .519 by Kruskal–Wallis) or IVC CI (*p* = .834 by Kruskal–Wallis) different among these three CKD groups.

Data for other relationships including physical examination and NT‐proBNP are shown in Table S1.

## 4 DISCUSSION

We examined the relationship between SCV CI and IVC CI in spontaneously breathing patients hospitalized with ADHF. Our data showed a significant correlation between SCV CI and IVC CI and determined cutoffs for SCV CI that were predictive of IVC CI cutoffs which suggest hypervolemia or hypovolemia, with reasonable sensitivity and specificity. This study verified the presumed relationship between clinically significant TR and IVC CI and showed a similar effect on SCV CI.

The correlation between SCV CI and IVC CI in our population is similar to other studies of spontaneously breathing patients, positioned supine at 30–45°. Our correlation (*R* = .65, *n* = 36) was similar to patients hospitalized with acute and chronic renal failure (*R* = .67, *n* = 95)^23^ and patients undergoing echocardiography (*R* = .69, *n* = 39).^22^ The relationship of SCV CI to IVC CI in patients undergoing echocardiography has been shown to be inferior when SCV CI was measured supine at 0°.^22^ Assessment of SCV CI, as with physical examination of JVP, is highly position‐dependent and should be performed supine at 30–45°.^22^, ^23^ On the other hand, there are no differences for IVC_max_ or IVC CI when measured at 0° versus 45°.^33^

Our SCV CI cutoff of <22% which best predicted an IVC CI cutoff <20% suggesting hypervolemia (sensitivity/specificity 72%), is similar to spontaneously breathing hospitalized patients with acute and/or chronic renal failure (SCV CI cutoff <22%, sensitivity/specificity 74%).^23^ Our SCV CI cutoff of >33% which best predicted an IVC CI cutoff >50% suggesting hypovolemia (sensitivity/specificity 78%), compared favorably to the SCV CI cutoff >39% (sensitivity/specificity 70%) in the aforementioned study.^23^

Our patients with at least moderate TR, compared to mild or no TR, had lower median SCV CI (12.4% vs. 25.3%) and median IVC CI (9.6% vs. 35.6%) indicating that clinically significant TR decreases venous collapsibility with respiration, which may lead to overestimation of relative intravascular volume. The relationship of SCV CI to TR had not been previously reported to our knowledge.

Shapira et al.^20^ reported that IVC CI was ≤50% in 26 of 38 patients with moderate to severe TR and in 8 of 28 patients with less than moderate TR. These data are consistent with the present findings of lower IVC CI with at least moderate TR in patients with ADHF. In a study of 96 patients with severe TR, those with signs of right‐sided congestive heart failure had lower IVC CI (mean ± SD: 11.2% ± 8.5%) than those without signs of right‐sided congestive heart failure (mean ± SD: 24.3% ± 14.1%, *p* = .001),^15^ indicating that IVC CI may vary with relative intravascular volume even in the presence of severe TR.

Our study showed significantly larger SCV_max_ (0.89 cm vs. 0.58 cm) and IVC_max_ (2.4 cm vs. 2.1 cm) with moderate to severe TR compared to less than moderate TR. The only other published data also showed larger IVC_max_ (2.4 cm vs. 1.9 cm, respectively) with moderate to severe TR compared to less severe TR.^20^

Overall, these findings indicate that SCV_max_, IVC_max_, and collapsibility with respiration may be independently affected by both relative intravascular volume and significant (at least moderate) TR. The presence of clinically significant TR may bias the interpretation of venous maximum diameter and collapsibility with respiration resulting in overestimation of relative intravascular volume.^10^

Because SCV CI and IVC CI were not significantly affected by the presence or absence of right‐sided intracardiac devices or CKD stage, our data suggest that the use of SCV CI as a surrogate for IVC CI in the assessment of relative intravascular volume may be applicable to a general population of patients with ADHF that have right‐sided intracardiac devices, and CKD Stages 2–4. SCV CI and IVC CI may also reflect RAP in patients with rate‐controlled atrial fibrillation/flutter.^27^

The limitations of our study are that this is a pilot study with a small but adequately powered sample size, and echocardiograms assessing the severity of TR were not obtained on the same day as SCV and IVC ultrasound.

In conclusion, SCV CI measured supine at 30–45° correlated well with paired IVC CI by ultrasound in non‐ventilated patients hospitalized with ADHF and may provide an alternative to IVC CI for assessment of relative intravascular volume. SCV or IVC collapsibility with respiration may therefore be a useful adjunct to other available clinical information in assessing and managing ADHF. Moderate to severe TR decreases SCV CI and IVC CI and may result in overestimation of relative intravascular volume.

## CONFLICT OF INTEREST

The authors declare that there is no conflict of interest.

## Supporting information

None.

## Data Availability

Data will be made available upon reasonable request.
