# Supplementary material for: Comparison of subclavian vein to inferior vena cava collapsibility by ultrasound in acute heart failure: A pilot study
Source: Clin Cardiol. 2021 Dec 21;45(1):51–9. doi: 10.1002/clc.23758 (PMC8799052; doi:10.1002/clc.23758)
Supplement: Supplementary file 1 — None. [file CLC-45-51-s001.docx]

**Supplemental TABLE 1 Clinical Comparisons**

| **CORRELATIONS** | | | | |
| --- | --- | --- | --- | --- |
| **Variable 1** | **Variable 2** | **Correlation coefficient (R)** | **Number** | **p-value** |
| JVP initial assessment near admission | Log_10_NT-proBNP | 0.59 | 33 | <0.001 |
| SCV CI | JVP | -0.42 | 36 | 0.012 |
| IVC CI | JVP | -0.42 | 36 | 0.010 |
| **GROUP COMPARISONS** | | | | |
| **Variable** | **Groups** | **Differences among groups** | **Number** | **p-value** |
| JVP | Estimated RAP 3, 8, or 15 mmHg per ASE guidelines ([9](#_ENREF_9),[10](#_ENREF_10)) | JVP values were higher for ASE category of 15 mmHg | 36 | 0.011  Kruskal-Wallace followed by Duncan test at p value of 0.05 |
| JVP | Moderate or greater TR, Less than moderate TR | JVP higher with moderate or greater TR | 33 | 0.008  Mann-Whitney |
| **CONTINGENCY TESTING OF NUMBERS IN EACH GROUP** | | | | |
| **Grouping 1** | **Grouping 2** |  | **Number** | **p-value** |
| Estimated RAP 3, 8, or 15 mmHg per ASE guidelines ([9](#_ENREF_9),[10](#_ENREF_10)) | Moderate or greater TR, Less than moderate TR | More patients with at least moderate TR had estimated RAP of 15 and fewer had estimated RAP of 3 compared to those with mild or no TR | 33 | 0.039  Log likelihood ratio test |

Abbreviations: ASE=American Society of Echocardiography; CI=collapsibility index; IVC=inferior vena cava; JVP=jugular venous pressure; NT-proBNP=N-terminal prohormone of brain natriuretic peptide; RAP=right atrial pressure; SCV=subclavian vein; TR=tricuspid regurgitation.

Non-significant relationships include: Severity of lower extremity edema (0 to 4+) with SCV CI, IVC CI, JVP, or Log_10_NT-proBNP; Log_10_NT-proBNP with ejection fraction, SCV CI, or IVC CI.
